# Microvascular Anastomosis in Practice: A Pilot Study on Microsurgical Training Efficiency

**DOI:** 10.3390/clinpract15050082

**Published:** 2025-04-22

**Authors:** Florin-Vlad Hodea, Andreea Grosu-Bularda, Andrei Cretu, Catalina-Stefania Dumitru, Vladut-Alin Ratoiu, Eliza-Maria Bordeanu-Diaconescu, Raducu-Andrei Costache, Razvan-Nicolae Teodoreanu, Ioan Lascar, Cristian-Sorin Hariga

**Affiliations:** 1 Department 11, Discipline Plastic and Reconstructive Surgery, Bucharest Clinical Emergency Hospital, University of Medicine and Pharmacy Carol Davila, 050474 Bucharest, Romania; florin-vlad.hodea@drd.umfcd.ro (F.-V.H.); andrei.cretu@drd.umfcd.ro (A.C.); catalina-stefania.dumitru@rez.umfcd.ro (C.-S.D.); vladut-alin.ratoiu@rez.umfcd.ro (V.-A.R.); raducu-andrei.costache@rez.umfcd.ro (R.-A.C.); razvan.teodoreanu@umfcd.ro (R.-N.T.); ioan.lascar@umfcd.ro (I.L.); cristian.hariga@umfcd.ro (C.-S.H.); 2 Clinic of Plastic Surgery and Reconstructive Microsurgery, Clinical Emergency Hospital of Bucharest, 014461 Bucharest, Romania; eliza.diaconescu@umfcd.ro

**Keywords:** microsurgery, training, microvascular, techniques, education

## Abstract

**Background:** Microsurgery is a demanding surgical field requiring precision and extensive training. There is a continued need for standardized training models to improve skill acquisition and efficiency in microvascular anastomosis. **Objectives:** This study aimed to assess whether a standardized microsurgery training protocol, focusing on technique-specific objectives, improves performance among beginner trainees. **Material and Methods:** A three-month, non-randomized cohort study was conducted with entry-level plastic surgery residents. Participants were assigned to either a control group, practicing without structured guidance, or a test group, using a predefined microsurgery curriculum. Skill performance was measured at baseline, three weeks, and three months using a modified University of Western Ontario Microsurgical Skills Assessment (UWOMSA) tool. **Results:** While both groups improved over time, the test group demonstrated significantly greater improvement at the three-month mark (mean score: 59 vs. 38; *p* = 0.00027). The structured training model promoted more consistent progress and superior microsurgical technique. **Conclusions:** A standardized training protocol significantly enhances microsurgical proficiency over time. These findings suggest value in structured, low-cost training models for microsurgical education. Limitations include the small sample size, use of non-living models, and a non-randomized design.

## 1. Introduction

Microsurgery is a highly specialized surgical field that employs precision instruments and magnification to perform intricate procedures on small structures such as blood vessels, nerves, and tissues. It has revolutionized the field of surgery by enabling surgeons to achieve outcomes that were previously unattainable. The success of microsurgery relies on a combination of factors, including meticulous decision-making, seamless logistical coordination, and refined execution. These elements work synergistically to optimize clinical outcomes, reflecting the incremental advancements that have been made in this demanding yet transformative discipline [1]. It can be applied in fields such as trauma, limb reconstruction, radiation injuries of the lymphatics, and breast reconstruction [2,3]. Technique-wise, besides dissection, i.e., neurorrhaphy, microsurgery frequently involves multiple microvascular anastomoses, in which both quality and timeframe can be improved [4]. Within any surgical specialty, there is a common association between prolonged operative time and complications such as flap failure, infection, dehiscence, deep vein thrombosis, and reintubation [5,6].

The objectives of this study were to evaluate different groups of surgeons involved in microsurgery at the entry-level stages of their microsurgical practice and evaluate their improvement in microvascular skills given a standardized set of acquirable skills and knowledge by applying a modified University of Western Ontario Microsurgical Skills Assessment (UWOMSA) instrument tool at different timeframes [7]. The UWOMSA instrument is an already established tool that evaluates microsurgical trainee skill based on a point system. However, it was adapted to expand its applicability to non-living models and early-stage trainees, with additional criteria targeting ergonomics, needle control, and lumen assessment. The modified version was reviewed internally for face validity by experienced faculty but has not yet undergone formal psychometric validation.

This study may impact microsurgery training programs, in which a well-defined set of skills may be taught to ease microsurgery integration timeframes. Additionally, the implementation of simulation-based training and mentorship programs can further enhance trainees’ proficiency, allowing them to practice these critical skills in a controlled environment before applying them in real surgical settings [8].

Despite the increasing adoption of microsurgical procedures, especially in reconstructive and supermicrosurgical fields, there remains a lack of standardized, validated training protocols tailored specifically for beginner surgeons. Many current programs are resource-intensive, lack consistency in technique emphasis, or fail to provide adequate assessment tools. This study aims to address this gap by evaluating a structured, reproducible training protocol for entry-level trainees using non-living models. Our objective was to determine whether implementing a predefined set of microsurgical techniques could lead to measurable improvements in microvascular anastomosis over a three-month training period.

## 2. Materials and Methods

A 3-month non-randomized cohort study was performed on plastic and reconstructive surgery residents with beginner-level experience in the field of microsurgery at the Clinical Emergency Hospital Bucharest, Romania. The inclusion criteria were resident surgeons in plastic and reconstructive microsurgery specialties, who were considered beginners (under 15 points) from the initial enrollment questionnaire and were able to train for two hours weekly, for three months, in the microsurgical laboratory of the Clinical Emergency Hospital, Bucharest. In addition, all participants underwent the same introductory microsurgery course beforehand, which involved knowledge of basic ergonomics, instrument handling, and simple suturing. The exclusion criteria were surgeons who were not able to undergo weekly spaced repetition or those who scored high in the initial microsurgery-expertise questionnaire. All participants consented to participate in this study.

This study was designed as a non-randomized cohort study due to limited access to participants with similar baseline microsurgical exposure during the same training period. While this approach may introduce selection bias, all participants were selected from the same institution and had comparable baseline skills, as verified by a standardized questionnaire. This pilot design allows for an initial evaluation of the training model’s feasibility and impact, with the goal of informing future randomized studies.

A questionnaire, as seen in Table 1, based on the number of microsurgeries performed, types of procedures, experience in the field with microsurgery tools and techniques, frequency of procedures, years of practice, training and education, outcome of surgeries, complexity of cases, and continuing education in microsurgery divided the categories into beginner, intermediate and advanced, with a threshold differentiation of 15 points, beginner within the interval of 10–15 points, intermediate between 16 and 30 points and advanced between 31 and 45 points. The scoring thresholds used to classify participants as beginner, intermediate, or advanced were developed through internal consensus among senior microsurgeons, based on training exposure and procedure complexity, and were applied consistently across all subjects. The final participants were residents in the second to fourth year of residency.

A standardized set of procedures was established, consisting of techniques that enhance speed, preparation, suturing, final result, needle and instrument handling, as well as ergonomics, with the same number of practice hours and the same reference material. Participants in the test group received a live, structured instructional session at the outset of the program, during which senior faculty members demonstrated the full sequence of microsurgical techniques that would subsequently be practiced in the laboratory. Moreover, an additional control group was established that only practiced through practice-based repetition, without applying the aforementioned reference material. The same scoring system was used for all groups at all times. All participants were evaluated from the same starting skill category, using the same microscope, instruments, and set-up. All procedures were performed on non-living models, ex-vivo chicken legs on femoral artery vessels of 2–3 mm diameter, end-to-end technique, using a „blue-blood” flow-through system as described by Zeng et al. [9]. Data were gathered from participants during each assessment period. To minimize bias, evaluators were blinded to group assignments during each assessment. Assessments were performed independently by experienced microsurgeons from senior authors. The findings were evaluated and reviewed by peers. The obtained results were analyzed in comparison with current medical papers found in the Web of Science, PubMed, and Google Scholar databases. PaperPal Preflight was used as a support tool for grammar and language correction as a pre-submission checker and for evaluation of this manuscript.

Data were collected based on an objective and subjective set of measurements using a modified score based on the UWOMSA instrument during initial practice and at three weeks and three months after training in both the control and experimental groups. Our study adapted the UWOMSA tool to better suit entry-level microsurgery trainees and non-living models by expanding its scope and specificity. Our study modified the UWOMSA tool, as seen in Table 2, to provide a more comprehensive and tailored evaluation framework for beginner-level microsurgical trainees using non-living models. Key additions include detailed metrics for task duration and preparation, such as ensuring perpendicular clamp placement and proper adventitial trimming. Suturing metrics were refined to emphasize precision and consistency, including needle placement, atraumatic needle passage, fluid knot tying with correct tension, and regular lumen checks. The final product criteria were expanded to assess patency, symmetric suture spacing, and proper outer appearance, avoiding stenosis or leakage. Additional metrics for ergonomic efficiency, such as maintaining correct posture and efficient instrument handling, were included to address the practical challenges of long procedures. These modifications ensure a reproducible, skill-focused assessment method that bridges gaps in microsurgery training for entry-level practitioners.

Based on a score system, as seen in the two expertise groups we created, the beginner group scored under 15 points, and the advanced group scored 16 points or above. Only beginners were included in this study.

The variables included in this study were microsurgery training, skill level based on points, and time frame. The variables were analyzed using Microsoft Excel. Independent *t*-tests were used to establish whether the null hypotheses were true (*p*-values significantly under 0.05). A repeated measures ANOVA was used to analyze the effect of time and group on skill acquisition across three timepoints (Initial, 3 weeks, 3 months).

## 3. Results

A group of eight participants was included in this study, with seven scoring 10 points and one scoring 12 points on the initial skill assessment, based on their beginner-level experience in microsurgery. These participants were then divided into a test group and a control group, each comprising four individuals. The main baseline characteristics were their initial skill assessment scores, which demonstrated comparability between the groups, ensuring a balanced starting point for evaluating the outcomes of the standardized training protocol.

To assess microvascular suturing efficiency, a modified version of the UWOMSA tool was applied with a set of 15 questions scoring 1, 3, or 5 points, with a possible minimum score of 15 points and a maximum of 75 points. Measurements were taken during the first training session, after 3 weeks, and after 3 months. The following were taken into account: anastomosis, preparation of the microsurgical field, suturing, anastomosis end-product, ergonomics of set-up, needle, and instrument handling.

During the initial baseline evaluation, the groups scored 26.5 and 26 points for the test, respectively, for the control group. The test group was subjected to microsurgical reference material detailing aspects of ergonomics, instrument handling, and basic and advanced microsurgical techniques. After 3 weeks, the average scores were 40 and 35 points for the test and control groups, respectively. Finally, after three months of training, the average results were 59 points for the test group and 38 points for the control group. Table 3 presents the results. For the evaluation of groups at different times, an independent *t*-test was performed, with results being statistically significant for the comparison of groups at the 3-month timeframe evaluation. The reported *p*-values reflect between-group comparisons at each specific timepoint using independent *t*-tests. No participants dropped out of this study, and all data points were complete across the three assessment periods.

The standard error of the mean (SEM) and 95% confidence intervals (CIs) were calculated for both the test and control groups at each evaluation timepoint. The test group demonstrated lower SEM values across all timepoints, with narrower Cis indicating more consistent performance. At the 3-month evaluation, the test group achieved a mean score of 59 (95% CI: 52.40 to 65.60), compared to the control group’s mean of 38 (95% CI: 30.91 to 45.09).

A repeated ANOVA was performed for analysis of individual progression. Mauchly’s test of sphericity indicated that the assumption of sphericity was met (*p* = 0.158), confirming the appropriateness. The repeated ANOVA revealed a significant effect of time on skill acquisition (*p* < 0.001), with mean scores increasing progressively across the three timepoints: 26.25 at the initial evaluation, 37.5 at 3 weeks, and 48.5 at 3 months. Post-hoc pairwise comparisons, corrected using the Bonferroni method (α = 0.017), identified significant differences between all pairs of timepoints (initial vs. 3 weeks, initial vs. 3 months, and 3 weeks vs. 3 months), indicating substantial and sustained improvement in microsurgical skills over the study period.

## 4. Discussion

This pilot study demonstrated that a structured microsurgical training protocol can significantly enhance skill acquisition in beginner-level trainees over a short timeframe. The use of a modified and standardized UWOMSA tool allowed objective tracking of progress, highlighting significant differences in performance between the test and control groups at the three-month evaluation. The observed improvements in task precision, time efficiency, and ergonomic posture underscore the value of structured, skill-specific microsurgical education.

Participants were not informed about the questionnaire during this study. After being provided with reference material, which aimed to improve different microsurgery skill categories, using specific techniques, with key objectives, as seen in Table 4, the groups were only required to practice microvascular anastomoses for two hours, weekly, over a period of three months, the test groups with newly acquired reference material, and the control groups practicing previously known skills. All participants shared a comparable baseline of microsurgical skill, namely, instrument familiarity, basic suture technique, and microscope handling, acquired during standard introductory training at the start of their residency.

For ergonomics, pre-surgical preparation details included room environment and set-up of non-living material, microscope, instruments, and sutures [10]. A key emphasis was placed on maintaining proper posture during procedures, with the correct microscope-to-eye level. Trainees were instructed to keep their shoulders relaxed and their spine straight, a posture that is crucial for minimizing back strain. Additionally, they were advised to rest their elbows and ulnar side of their hands on a stable surface to enhance precision and reduce fatigue [11,12].

For preparation, the test participants were instructed first to dissect two–three centimeters of the vessel. After the vessel was prepared, the timer was started from the time of sectioning. Next, for clamp placement, they were instructed to use a double microvascular clamp placed perpendicular and symmetric between the two vessel ends, approximating them in a tensionless fashion, with the lumen edges barely in contact [13]. Dilation was achieved mechanically using dilator forceps in two directions, followed by thorough washing with heparinized solution, without excessive force, which may injure the intimal layer. Finally, preparation included adventitia trimming, for which the participants were instructed to pull the adventitia over and cut it parallel to the vessel end. In this way, a length of 2–3 mm of adventitia is trimmed on each side of the anastomosis, avoiding excessive removal of the adventitia and insufficient adventitiectomy, which may result in catching the adventitia inside the lumen [14,15].

For instrument handling, the test participants were encouraged to place the instruments in the same constant place, allowing easier access without breaking the microscope focus. For a right-handed person, this usually means placing the micro-forceps to the left of the field, the needle holder to the right of the microsurgical field, and the scissors either to the right or distal to the participant. While this position is not mandatory, some form of constant setup is recommended based on personal preferences. Thus, the microscope focus is not interrupted, saving time and preventing not only fatigue to the surgeon’s neck and shoulder, but also eye strain from repeatedly adjusting the macroscopic views to the microscope field. Maintaining this ergonomic arrangement can significantly enhance the efficiency of the procedure, allowing smoother transitions between instruments and minimizing the risk of errors during delicate maneuvers [16,17,18].

**Figure 1:**
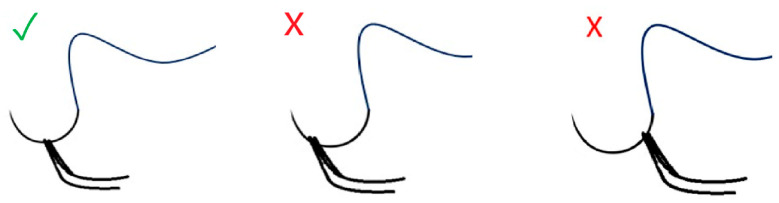
Preferable needle holding position for better control is with the needle holder just distal to the midpoint of the needle. The symbol “√” marks the correct position, while the symbol “×” indicates the incorrect position for needle holding.

For needle insertion into the vessel wall and subsequent needle passage, the following methodology was used. The needle was instructed to be held just beyond the midpoint of its curvature to obtain an adequate grip to effortlessly penetrate the vessel wall, as shown in Figure 1. Grabbing it too distally may cause discomfort by compensating with exaggerated pronation during suturing, and grabbing it too proximally may cause a weak grip on the needle, as well as increase the chance of penetrating the back wall, given the direction of the curvature of the needle. Subsequently, it was recommended that forceps be used to apply counterpressure when penetrating the vessel wall. The needle should be advanced to a depth corresponding to the thickness of the vessel wall while slightly everting the distal edge of the vessel, thereby facilitating the completion of the needle’s passage [18].

**Figure 2:**
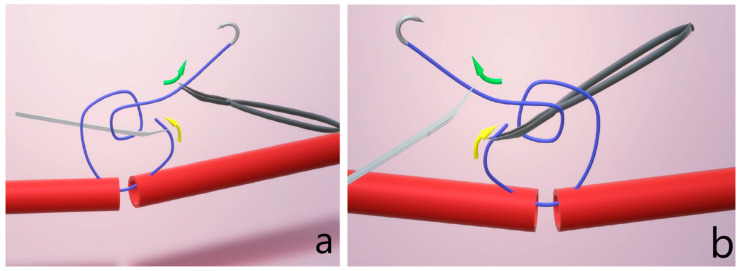
Ambidextrous knot tying for suturing, (**a**) using the left hand holding microforceps to pull the short end; (**b**) using the right hand holding the needle holder to pull the short end. Yellow arrows depict the direction of pull of the short end, green arrows depict the direction of pull of the long end.

The training program focused on movement during anastomosis. One such skill is ambidextrous knot tying, which can significantly improve surgical efficiency. This reduces the duration needed to securely fasten sutures while also minimizing the overlapping of a surgeon’s hands, which can often obstruct the field of view, as seen in Figure 2 [12]. Moreover, ambidextrous knot tying offers flexibility in positioning either the forceps or needle holder to face the shorter end of the suture. This adaptability is particularly advantageous in complex anatomical situations with tight spaces, where a specific direction of suture pull may be more effective or necessary for optimal results.

Manipulation of the long end of the thread during microvascular suture tying, while not a distinct technique, plays a crucial role in facilitating the creation of a knot. To optimize this process, the long end of the thread should be grasped at a length approximately two to three times that of the short end [18,19]. This segment of the thread is then maneuvered downward, aligning with the orientation of the short end. By doing so, the inherent spring tension and inherent memory of the thread are leveraged, aiding the effortless formation of loops [18].

**Figure 3:**
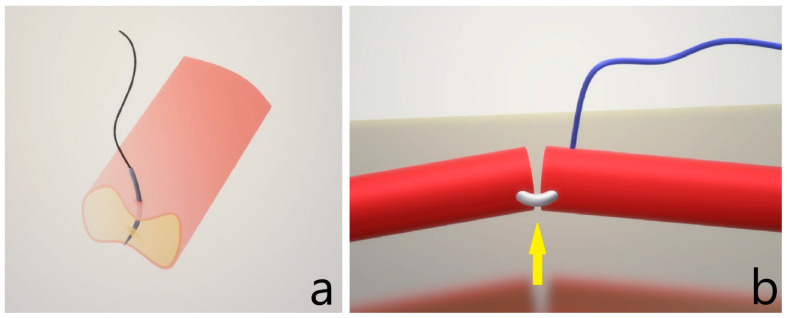
Inadvertent catch of the back wall, figure (**a**) back wall bite with only partial wall catching, harder to recognize; (**b**) back wall bite with “steel sign” seen by flipping the vessel on its back wall, as indicated by the yellow arrow.

In addition, it is essential to assess the lumen during and after each passage by performing lumen washing alongside suturing. This step not only removes any debris but also further improves the clarity of the microsurgical field, ensuring a more precise and safer procedure [18,20]. In our study, since the participants were sutured without the aid of a secondary surgeon, they were instructed to evaluate the lumen with a dilator or check in case of a back wall suture. It was advised to flip the vessel over to inspect for either a ‘steel sign’ or a ‘suture sign’ if a back-wall suture was suspected, as seen in Figure 3. This examination helps confirm whether the suture has inadvertently caught the back wall of the vessel, allowing for timely correction and ensuring anastomosis integrity [10].

**Figure 4:**
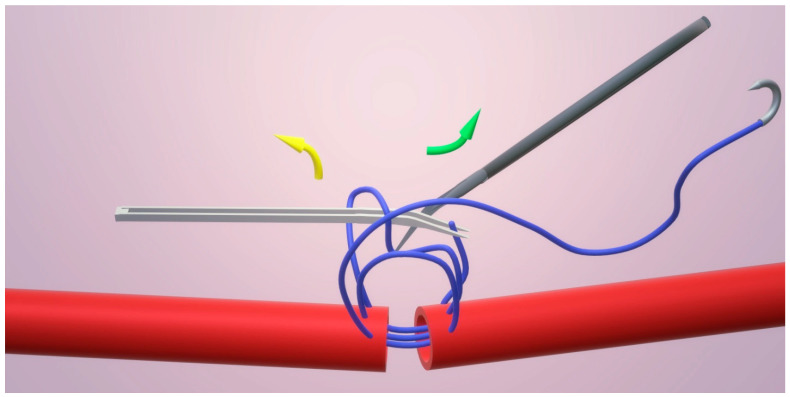
Open loop/continuous interrupted technique in which lumen is always checked for back wall bites. Yellow arrow depicts direction of pull of short end, green arrow depicts direction of pull of long end.

An open-loop technique or continuous interrupted suture technique controls lumen obstruction throughout suturing, as shown in Figure 4. It increases the speed of the procedure while ensuring the integrity of the vessel lumen. This effectively avoids back-wall stitch and guarantees evenly spaced sutures. In this approach, a continuous running suture style was employed following the placement of the stay sutures. The key feature of the open-loop is the creation of loops that remain open during the suturing. These loops were individually cut, and each knot was tied progressively. This method allows continuous monitoring of the lumen throughout the procedure, ensuring that the suture does not catch the back wall of the vessel. Additionally, the open-loop facilitates a more efficient suturing process, as the surgeon can quickly and accurately place sutures with consistent spacing, which is vital for the patency and strength of the anastomotic site. Another advantage is that it allows the microsurgeon to know the position of the needle permanently [21,22,23,24].

An additional technique that enhances control and precision within the microsurgical field involves strategic placement or “parking” of the needle for the next stitch within the vessel wall. This method allows the surgeon to determine the exact position of the needle at all times. By employing this technique, where multiple passes of the needle are made within the wall, the risk of inadvertently capturing the back wall of the lumen (a “back wall bite”) is significantly reduced [10,25].

Moreover, in situations where the microsurgeon chooses not to use the ‘inside wall parking’ method for the needle, it is advisable to park the needle in a constant area, preferably in alignment with the direction of the short end of the suture [26,27]. This alternative approach not only aids in maintaining a clear view of the position of the needle but also facilitates the creation of loops and assists in organizing the microsurgical field, thus facilitating smoother and more efficient suturing.

Management of suture ends is critical for preventing thrombosis and ensuring patency. While specific lengths are not universally prescribed, the consensus is that suture ends should be cut short enough to minimize the risk of thrombosis but long enough to maintain knot integrity [28].

Analysis of the test and control groups was performed across three separate timeframes: the initial evaluation, a follow-up assessment conducted after a duration of three weeks, and a concluding evaluation that took place at the three-month mark. The absence of a violation of the sphericity assumption ensures the robustness of the repeated measures ANOVA results. The significant differences observed between all timepoint pairs in the post-hoc analysis underscore the efficacy of the training intervention, with substantial gains noted even between the 3-week and 3-month evaluations. These results suggest that the intervention fosters sustained skill development, likely due to the structured and progressive nature of the training.

**Figure 5:**
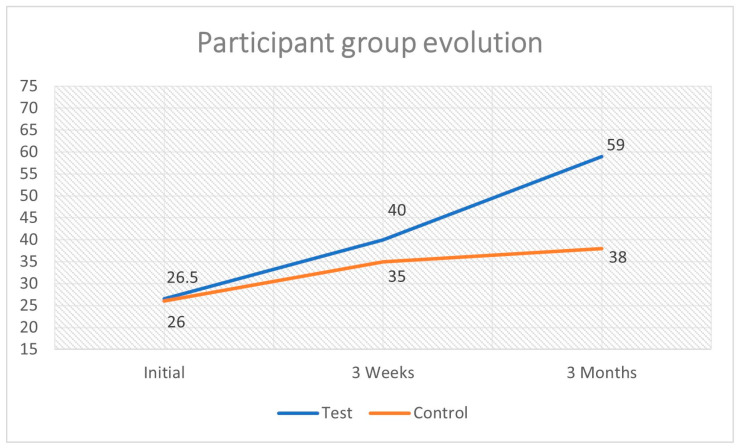
Participant group evolution.

To ascertain whether there was a statistically significant difference in the performance outcomes between the two groups at each of these evaluation stages, an independent *t*-test was employed as the analytical method of choice. During the initial evaluation phase, the test group achieved an average score of 26.5, in contrast to the control group, which began with a score of 26. This nearly identical baseline measurement revealed that both groups commenced their evaluations under similar conditions, a factor that is essential for validating the comparisons that will be made subsequently. Importantly, no statistically significant differences were observed between the two groups at this initial starting point, as indicated by the *p*-value of 0.875, which suggests a high level of similarity in their performance levels. Following the three-week period, the test group exhibited a noticeable increase in their average score, rising to 40, while the control group achieved a score of 35. Although it is evident that both groups made improvements in their scores, the test group displayed a slightly higher increase in their performance. However, this difference lacked statistical significance, as shown by the *p*-value of 0.0941, which indicates that the variations observed may not be robust enough to be considered meaningful. Upon reaching the evaluation point at three months, the distinctions between the performances of the two groups became significantly more pronounced and easier to identify. This considerable divergence in scores suggests that the microvascular technique employed within the test group not only provides immediate short-term benefits but also facilitates more enduring and long-term advantages when compared to the outcomes of the control group. The statistical evidence in this case is compelling, with a *p*-value of 0.00027, indicating a highly significant difference. Furthermore, the expanding gap in performance levels, as seen in Figure 5, observed between the two groups from the three-week assessment to the three-month evaluation, strongly emphasizes the effectiveness of the test intervention over an extended period of time.

The CI indicates the enhanced performance and consistency achieved by the test group, particularly at the 3-month evaluation. The narrower intervals and lower SEM values in the test group suggest that the standardized training protocol not only improved skill acquisition but also minimized variability among participants. In contrast, the control group displayed wider CIs, indicating less consistent progress and greater variability in outcomes. These results show the effectiveness of the training intervention in facilitating reliable and measurable improvements in microsurgical skills. However, the relatively small sample size likely influenced the observed variability, warranting further investigation with larger cohorts to validate these findings. The non-randomized design and small sample size are acknowledged limitations of this study, inherent to its exploratory and pilot nature. Post hoc power analysis, that a larger sample of participants per group would ensure sufficient power. Despite the small cohort, the results showed a statistically significant improvement in the test group after three months (*p* = 0.00027). Future multicentric studies will address these issues by employing randomization and larger cohorts to validate these findings.

Developing a low-cost and efficient microsurgery training program is crucial for enhancing surgical skills, particularly in resource-limited settings. Even without a microsurgical laboratory, different settings have been described, such as using a smartphone camera as a magnification tool, which eliminates the need for expensive microscopes. This approach allows for practice in vessel dissection and suturing, making it a viable option for early exposure to microsurgical techniques [29]. While these low-cost solutions significantly enhance accessibility to microsurgery training, they may not fully replicate the experience and precision of the high-end equipment used in advanced surgical procedures. However, they provide a crucial stepping stone for aspiring surgeons, particularly in under-resourced regions, by offering foundational skills and practice opportunities. As technology advances, the integration of virtual reality and augmented reality could further bridge the gap between low-cost training models and high-fidelity surgical environments [30,31]. In our study, we aimed to replicate the fidelity of blood hemodynamics as closely as possible to real-life clinical scenarios. Nevertheless, the literature has proven that pre-clinical, well-established microsurgery training programs can enhance the skill set of beginner microsurgeons [32].

While previous studies have established the benefits of repetitive microsurgical training, few have assessed the impact of targeted technique instruction on entry-level trainees using non-living models. Our findings align with those of Sert et al. (2023) and Dave et al. (2022), who reported skill enhancement following microsurgical simulation training. However, the present study is unique in its use of a modified evaluation tool tailored for beginners and its integration of low-cost, reproducible training materials applicable to limited-resource environments [21,31].

Despite these promising results, this study has certain limitations. First, training and assessment were conducted on non-living models, which are not always transcribable to real-life scenarios. In addition, while non-living chicken leg models are widely used, there may be differences in vessel elasticity, handling, or other characteristics compared to living human vessels. Secondly, this study was conceived as non-randomized, with few participants in each group and a short timeframe, potentially inducing selection bias. The small sample of participants can be attributed to the difficulty in finding a large number of participants with the same skill level and with the same microsurgical background, which can be solved through a multicentric study. Moreover, while the questionnaire served effectively for baseline stratification, its scoring system has not been externally validated and should be refined and standardized in future studies. The modified UWOMSA instrument, though effective, might not cover all aspects of microsurgical skills, and, more importantly, does not discriminate between the importance of each criterion of the assessment tool [33]. These limitations suggest that further research and real-world applications are necessary to fully validate these techniques. Additionally, future studies should consider a larger sample size and a randomized controlled design to enhance the reliability of the findings. Moreover, incorporating diverse training environments and varying levels of experience among participants could yield more comprehensive insights into the effectiveness of assessment methods.

## 5. Conclusions

This study demonstrates that structured microsurgery training protocols can produce significant improvements in microsurgical performance among entry-level trainees. The integration of ergonomic instruction, refined suturing techniques, and consistent feedback through a modified assessment tool contributed to superior outcomes over three months. These findings support the incorporation of standardized training models into microsurgical curricula, particularly in settings where resources and trainee time are limited.

The development of low-cost training environments further highlights the potential for wider access to microsurgical education, particularly in resource-limited settings. Although limitations exist, including the non-randomized nature of this study and the use of non-living models, the results provide promising insights for optimizing training methodologies for future surgeons. While this pilot study provides valuable insights into microsurgical training methodologies, future research with randomized designs and larger sample sizes is necessary to confirm its findings. These findings support the continued integration of standardized microsurgery training models into medical education, and further research is needed to validate these approaches in real-life clinical settings.

## Figures and Tables

**Table 1:** Microsurgical level questionnaire.

Question	1 Point	3 Points	5 Points
Number of microsurgeries performed	Less than 50	50–150	More than 150
Types of microsurgeries performed	Simple/none	Intermediate	Complex
Frequency of performing microsurgeries	Less than once a month	Monthly	Weekly
Experience with specific microsurgical tools and techniques	Basic/none	Moderate	Advanced
Years of experience in microsurgery	Less than 5 years	5–10 years	More than 10 years
Microsurgery training and education	Self-taught	Microsurgery courses	Microsurgery fellowship
Experience in microsurgical research	None	Participated in research studies	Led research studies
Outcome of surgeries (rate your success rate from 1 to 5)	Under 70% or not applicable	>70% success rate in the past year	>90% success rate in the past year
Case difficulties	Low difficulty (end-to-end anastomoses or basic nerve repairs)	Moderate complexity cases (pedicle dissections or single free flaps)	High-risk or challenging cases (composite flaps, perforator flaps, or multiple anastomoses)
Continuing education in microsurgery	Rarely or never attends	Occasionally attends workshops/seminars	Regularly attends

**Table 2:** Modified UWOMSA tool for evaluation of microvascular skills.

Task	1 Point	3 Points	5 Points
Duration to Complete Task	>25 min	15–25 min	<15 min
Preparation: Clamp Placement	Oblique angled, jaws not grasping the entire vessel, tensioned anastomosis	Slightly angled, tensionless anastomosis	Perpendicular with even distribution of jaws placement, tensionless anastomosis
Preparation: Dilatation and adventitia cleaning	Forgets	Rough dilation, adventitia not cleaned	Gentle dilation with minimal force and even adventitia excision
Suturing: Needle Placement	Back wall stitch or partial only wall placement	Oblique placement through all wall layers	Perpendicular placement through all wall layers
Suturing: Needle Passage	Rough passage with damage	Rough passage without damage	Gentle, atraumatic and fluid movement
Suturing: Knot Tying	Rough tying with diminished tension within the knots	Rough tying with vessel wall dragging, but correct tension	Fluid motion of tying, correct knot tension
Suturing: Lumen Check	Forgets to check more than three times	Forgets to check 1 to 3 times	Always checks
Suturing: Movement at Anastomosis	Overlap of hands with obstruction of field	No field obstruction, but struggling for fluent motions	Ease of motion with no field obstruction
Final Product: Outer Appearance	Inversed or kinked anastomosis	Areas of vessel wall overlapping	Slightly everted, aligned straight
Final Product: Patency	Completely obstructed, back wall stitch	Mild stenosis or leakage	Even-to-vessel caliber anastomosis, no stenosis, no leakage
Final Product: Suture Ends	Unevenly, in the lumen	Unevenly outside of the lumen	Even, outside of the lumen
Final Product: Suture Spacing	Asymmetric both spacing towards the vessel ends and between sutures	Spaced, but at irregular intervals	Symmetric spacing
Ergonomic Efficiency and Posture	Posture is hunched, finds difficulty in every step of the preparation and anastomosis	Breaks correct posture sometimes to accommodate preparation and anastomosis	Correct posture throughout preparation and anastomosis
Needle handling	Loses track of both needle and thread at least multiple times	Lost track of needle once	Permanent control of the needle location
Instrument handling	Constant improper usage of microsurgical instrument, e.g., dilator for grabbing, scissors for dilating	Sometimes uses incorrect instrument	Correct manipulation of instruments

**Table 3:** Training results of test and control groups, initially, at 3 weeks and after 3 months.

Evaluation Timepoint	Test Group (Mean ± SEM) [95% CI]	Control Group (Mean ± SEM) [95% CI]	Test Group (Mean ± SD)	Control Group (Mean ± SD)	*p*-Value (*t*-Test)
Initial evaluation	26.5 ± 1.50 [22.78–30.22]	26 ± 2.65 [18.21–33.79]	26.5 ± 3	26 ± 5.3	0.8748
Evaluation after 3 weeks	40 ± 2.38 [32.28–47.72]	35 ± 0.82 [33.07–36.93]	40 ± 4.8	35 ± 1.6	0.0941
Evaluation after 3 months	59 ± 1.83 [52.40–65.60]	38 ± 2.08 [30.91–45.09]	59 ± 3.7	38 ± 4.2	0.00027

**Table 4:** Summary of microsurgical skills and training objectives for the experimental group.

Skill Category	Specific Techniques Taught	Key Objectives
Ergonomics and setup	Proper posture maintenance (relaxed shoulders, straight spine), resting elbows, and optimizing microscope-eye alignment to reduce fatigue.	Reduce surgeon fatigue and enhance precision during procedures.
Vessel preparation and handling	Adventitia trimming, symmetric and tension-free clamp placement, gentle lumen dilation.	Improve preparation efficiency and minimize vessel damage.
Suturing	Open-loop suturing method to ensure lumen patency, ambidextrous knot tying for efficiency and avoid hand overlapping, and maintaining consistent suture spacing.	Optimize suturing speed and reduce complications like lumen obstruction.
Needle handling and insertion	Correct needle gripping, atraumatic passage through vessel walls, and applying counterpressure to prevent back-wall bites.	Enhance control during needle manipulation and avoid errors.
Instrument handling and workflow	Standardized instrument positioning for easier access, minimizing interruptions in microscope focus, and promoting smoother transitions.	Streamline workflow for consistent and efficient instrument use.
Final product assessment	Assessing vessel patency, avoiding stenosis, ensuring even suture spacing, and visually confirming lumen integrity (e.g., flipping vessel to detect ‘steel sign’).	Ensure high-quality anastomosis with minimal errors and obstructions.

## Data Availability

Processed data are contained within the article. The raw dataset will be available on request from the authors.
